# Oxygen gradient ektacytometry does not predict pain in children with sickle cell anaemia

**DOI:** 10.1111/bjh.17975

**Published:** 2021-12-03

**Authors:** Amina Nardo-Marino, Jesper Petersen, John N. Brewin, Henrik Birgens, Thomas N. Williams, Jørgen A. L. Kurtzhals, David C. Rees, Andreas Glenthøj

**Affiliations:** 1 Department of Haematology, Centre for Haemoglobinopathies, Copenhagen University Hospital (Rigshospitalet), Copenhagen; 2 Department of Immunology and Microbiology, Centre for Medical Parasitology, University of Copenhagen, Copenhagen, Denmark; 3 Department of Haematological Medicine, King’s College Hospital, London; 4 School of Cancer and Pharmaceutical Sciences, King’s College London, London, United Kingdom; 5 KEMRI/Wellcome Trust Research Programme, Kilifi, Kenya; 6 Department of Medicine, Imperial College London, London, United Kingdom; 7 Department of Clinical Microbiology, Copenhagen University Hospital (Rigshospitalet), Copenhagen, Denmark

**Keywords:** sickle cell anaemia, red blood cell deformability, clinical trials, hydroxycarbamide, α-thalassaemia

## Abstract

The loss of red blood cell (RBC) deformability in sickle cell anaemia (SCA) is considered the primary factor responsible for episodes of acute pain and downstream progressive organ dysfunction. Oxygen gradient ektacytometry (Oxygenscan) is a recently commercialised functional assay that aims to describe the deformability of RBCs in SCA at differing oxygen tensions. So far, the Oxygenscan has been evaluated only by a small number of research groups and the validity and clinical value of Oxygenscan-derived biomarkers have not yet been fully established. In this study we examined RBC deformability measured with the Oxygenscan in 91 children with SCA at King’s College Hospital in London. We found a significant correlation between Oxygenscan-derived biomarkers and well-recognised modifiers of disease severity in SCA: haemoglobin F and co-inherited α-thalassaemia. We failed, however, to find any independent predictive value of the Oxygenscan in the clinical outcome measure of pain, as well as other important parameters such as hydroxycarbamide treatment. Although the Oxygenscan remains an intriguing tool for basic research, our results question whether it provides any additional information in predicting the clinical course in children with SCA, beyond measuring known markers of disease severity.

## Introduction

Sickle haemoglobin (haemoglobin S, HbS) is a structural variant of haemoglobin A (HbA). Sickle cell disease (SCD) refers to any symptomatic condition caused by HbS polymerisation within red blood cells (RBCs), with the most common and severe condition, sickle cell anaemia (SCA), resulting from homozygosity for the sickle mutation (HbSS). SCA is characterised by recurrent episodes of acute illness, chronic haemolytic anaemia and organ damage, as well as a significant reduction in life expectancy.^1^

When the oxygen tension is low, HbS polymerises causing the RBCs to become rigid and distorted—so-called *sickling*. Sickled RBCs are less deformable in the microcirculation, causing microvascular occlusion and haemolysis. The loss of RBC deformability in SCA is considered the primary factor responsible for episodes of acute pain and downstream progressive organ dysfunction.^2, 3^ An increased number of dense RBCs (DRBCs) has been shown to correlate with several complications in SCA, such as skin ulcers, priapism and renal dysfunction.^4^ Furthermore, decreased RBC deformability in SCA renders the cells more fragile and enhances the risk of haemolysis.^5^

In recent years, there has been increasing focus on SCD worldwide with many new treatments being proposed.^6^ There has been a surge in the number of clinical trials involving individuals with SCD and with these, a call for the development of new biomarkers to help predict disease severity and assess treatment response, outcomes that have traditionally been evaluated through patient reports.

One such proposed biomarker is RBC deformability. Ekta-cytometry is the gold standard for measuring RBC deformability. The method relies on laser diffraction analysis and allows for a direct measurement of alterations in the deformability of RBCs at 37°C under a predetermined shear stress.^7^ Ektacytometry of SCA RBCs, however, is complicated by the high proportion of rigid and sickled cells interfering with the elliptical diffraction pattern.^8^

Measuring deformability in SCA RBCs as a function of oxygen tension was first described by Sorette *et al*. in 1987. ^9^ Recently, oxygen gradient ektacytometry has been commercialised as the Oxygenscan,^10,11^ a functional assay that aims to describe the deformability of RBCs in SCA at differing oxygen tensions. RBC deformability is characterised as an elongation index (EI) under normal oxygen tensions (EI_max_) and during a hypoxic state (EI_min_). From these measurements a so-called point of sickling (POS), the patient-specific oxygen tension at which HbS polymerisation is thought to begin, is determined. So far, the Oxygenscan has been evaluated only by a small number of research groups and limited data on the method have been published.^10–16^ Thus, the validity and clinical value of Oxygenscan-derived biomarkers have not yet been fully established.

In this study we aimed to investigate the relationship between Oxygenscan-derived biomarkers and two established markers of disease severity in SCA: haemoglobin F (HbF) and co-inherited α-thalassaemia. A second aim of our study was to examine RBC deformability measured with the Oxygenscan in children with SCA treated with or without hydroxycarbamide (hydroxyurea, HU). Finally, we investigated whether Oxygenscan-derived biomarkers could be used to predict clinical outcomes, such as sickle cell pain and risk of stroke, as indicated by transcranial Doppler (TCD) velocities within the cerebral vessels.

## Methods

### Study population

We included a random selection of children aged 0–16 years with a confirmed diagnosis of SCA (HbSS) from the paediatric SCD clinic at King’s College Hospital in London. Children taking HU were only included if treatment had been initiated more than three months prior to recruitment and the dose was stable. Children were excluded if they had received one or more blood transfusions within three months of study recruitment.

### Ethical considerations

The National Health Service (NHS) Research Ethics Committee granted ethical approval for this study (ref: 18/LO/1566). Written informed consent was obtained from all children aged 16 years, prior to recruitment and sample collection. For children under the age of 16 years, a parent or guardian provided written informed consent. Additionally, children aged 11 to 15 years provided written assent.

### Oxygenscan analyses

Ethylenediamine tetra-acetic acid (EDTA) blood samples were collected by venepuncture on the day of study recruitment. Samples were stored and transported at approximately 4°C, from King’s College Hospital, London to Copenhagen University Hospital. All samples were analysed between 24 and 48 h of sampling using the Oxygenscan module of the Laser Optical Rotational Red Cell Analyser (Lorrca, RR Mechatronics, Zwaag, The Netherlands).

For each sample, a standardised number of RBCs (200 × 10^6^) were suspended in 5 ml of polyvinylpyrrolidone (PVP) buffer (osmolarity: 282-286 mOsm/kg, pH: 7·35-7·45, viscosity: 27·5-32·5 mPa) at room temperature (22°C), according to the manufacturer’s instructions.^10^ The temperature of the Oxygenscan cylinder was fixed at 37°C. For each analysis, 2 ml of the RBC–PVP suspension was injected into the Oxygenscan cylinder using a syringe. The machine was turned on for a minimum of 30 min before any study sample analyses, during which an Oxygenscan analysis of a nonsickle control sample was carried out to warm up the machine. This initial analysis acted as quality control, as a control sample should not sickle nor yield decreased EI_max_. A test was only repeated if recurrent drop outs in the measurement of EI occurred.

One Oxygenscan cycle comprises a round of deoxygenation (approximately 1300 s) facilitated by the inlet of nitrogen, making the pO_2_ in the system slowly drop from around 160 mm Hg to 20 mm Hg. This is followed by a round of reoxygenation (approximately 280 s) by passive diffusion of ambient air.^11^ The Oxygenscan produces a range of parameters, the three most informative being EI_max_, EI_min_ and POS.^10^ EI_max_ is the EI measured at normal oxygen tensions, representing deformability of the RBC population under conditions that are similar to those in the arterial circulation. EI_min_ is the EI measured at very low oxygen tensions (≤20 mm Hg), representing deformability of the RBC population under conditions that are similar to those in the postcapillary venules. POS is the point at which a more than 5% decrease in EI is observed, representing the individual oxygen tension at which sickling should theoretically begin.

Negative values of EI have previously been described in ektacytometry analyses of SCA RBCs.^8^ This phenomenon is thought to be caused by RBCs not aligning with the fluid flow (e.g. irreversibly sickled cells), with a negative value reflecting the applied image processing protocol and EI calculation. Any negative EI values were included in our data analyses.

### Haematological parameters

Samples for determination of HbF fractions, haemoglobin (Hb), markers of haemolysis, glucose-6-phosphate dehydrogenase (G6PD) activity and co-inherited α-thalassaemia were collected on the same day as the Oxygenscan sample and analysed routinely at the King’s College Hospital laboratory. HbF percentage (%HbF) was determined by high-performance liquid chromatography (HPLC) using a Variant II (BioRad Laboratories, Watford, UK). G6PD activity was tested with a direct enzyme activity assay. Samples were screened for the following common α-thalassaemia deletions using a multiplexed gap polymerase chain reaction (PCR) assay: 3·7 Kb, 4·2 Kb, 20·5 Kb, SEA, MED and FIL.^17^

### Data collection

Medical records were examined and information regarding HU treatment, as well as visits to the emergency department and hospital admissions in the previous two years, were recorded for all children. A painful crisis was defined as any acute sickle cell pain resulting in a visit to the emergency department or hospital admission. Furthermore, all children and their parents or guardians were asked about sickle cell pain frequency in the past 12 months, including painful episodes that did not lead to emergency department visits or hospital admissions. Self-reported frequency of pain was recorded as: daily, weekly, monthly, yearly or never.

For all children more than two years of age, results from their most recent TCD scan were recorded. Most children had a scan performed on the same day as the Oxygenscan sample was collected, although some had their latest scan up to nine months prior to study inclusion. Time-averaged means of the maximum velocities were recorded for all intracranial vessels using standard protocols^18^ and the highest velocity for each child (TCD_max_) was used in further analyses.

### Statistical methods

Means, standard deviations (SD) and percentages were calculated. Linear correlations were calculated using Pearson’s correlation and groups were compared using a twosided t-test or ANOVA. Continuous outcomes were examined using simple and multiple linear regression. Categorical outcomes were analysed using simple and multiple logistic regression. Ordinal logistic regression was used when appropriate. Multiple regressions were adjusted for potential confounding factors, such as age, %HbF, Hb and co-inherited α-thalassaemia. Missing values for %HbF and Hb were imputed for multiple predictor analyses. Imputed values were not used in analyses where %HbF was the outcome variable. Significance level was defined as *P* < 0·05. All statistical analyses were performed in Stata V16·1 (StataCorp, Timberlake, NC, USA).

## Results

### Study population

A total of 91 children with HbSS were recruited from January 2019 to January 2020. Baseline characteristics are summarised in Table I.

### Modifiers of disease severity

#### Haemoglobin F

**Fig 1:**
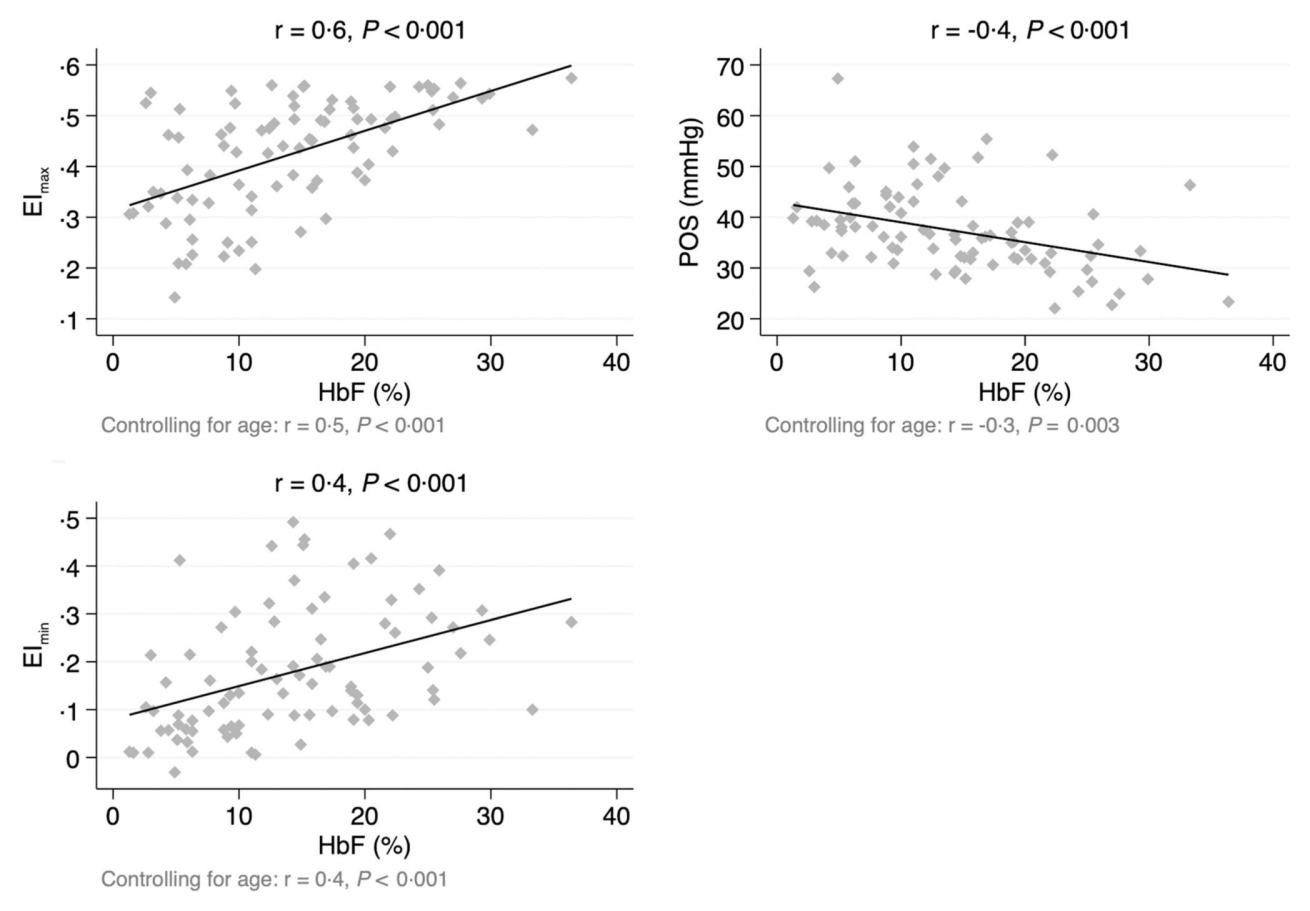
Linear correlation between Haemoglobin F percentage and Oxygenscan-derived biomarkers. EI, elongation index; HbF, haemoglobin F; POS, point of sickling; r, correlation coefficient.

HbF fractions were available for 86 children. Linear correlation graphs between %HbF and Oxygenscan-derived biomarkers are shown in Fig 1. EI_max_ and EI_min_ both demonstrated a significant moderate positive correlation with %HbF (EI_max_: *r =* 0·6, *P* < 0·001; EI_min_: *r =* 0·4, *P* < 0·001). POS demonstrated a significant moderate negative correlation with %HbF (r = —0·4, *P* < 0·001). Results were similar when controlling for age (Fig 1).

#### Co-inherited α-thalassaemia

Data on α-thalassaemia genotype were available for 87 children. Only the 3·7-kb deletion was detected: 33 children were heterozygous, 7 children were homozygous and 47 children had no deletions. Oxygenscan-derived biomarkers according to α-thalassaemia genotype are summarised in Table II. POS was significantly lower in children with hetero- or homozygous α-thalassaemia compared to children with no deletions (P = 0·006). EI_max_ was higher in children with hetero- or homozygous α-thalassaemia (P = 0·04), whereas results for EI_min_ showed higher values in children with heterozygous α-thalassaemia compared to children with no deletions, but lower values in children with homozygous α-thalassaemia. Results for EI_min_ were not statistically significant.

Multiple linear regressions were calculated to predict Oxygenscan-derived biomarkers based on α-thalassaemia genotype, controlling for age, %HbF and HU treatment. A significant association was found between EI_max_ and α-thalassaemia, and results suggested that EI_max_ was 0·7 SD higher in children with co-inherited α-thalassaemia compared to children with no deletions (P < 0·001). A significant association was also found for EI_min_, and results suggested that EI_min_ was 0·3 SD higher in children with co-inherited α-thalassaemia (P = 0·03). Finally, a significant association was found for POS, and results suggested that POS was 5·5 mm Hg lower in children with co-inherited α-thalassaemia (P < 0·001).

#### Glucose-6-phosphate dehydrogenase deficiency and markers of haemolysis

G6PD activity was tested in 86 children, nine of whom were found to be deficient. When comparing Oxygenscan-derived biomarkers in children with G6PD deficiency to children with normal G6PD activity, there was no significant difference in EI_max_, EI_min_ or POS (see Table III).

Linear correlations between Oxygenscan-derived biomarkers, Hb, bilirubin, lactate dehydrogenase (LDH) and reticulocyte percentage are presented in Table IV. There was a significant positive linear correlation between EI_max_ and Hb, and a significant negative linear correlation between POS and Hb. There was a significant negative correlation between EI_max_ and all three markers of haemolysis, whereas EI_min_ only showed a significant negative correlation with bilirubin. POS showed a significant positive correlation with bilirubin and LDH, but not with reticulocyte percentage.

### Hydroxycarbamide treatment

A total of 43 children were receiving HU treatment. Mean age in the HU group was 9·6 years (range: 2-16 years), compared to 7·3 years in the non-HU group (range: 7 months to 16 years). %HbF was higher in children treated with HU compared to children treated without HU (mean: 16·3% compared to 12·1%). Oxygenscan-derived biomarkers in children treated with HU compared to children treated without HU are summarised in Table V. EI_max_ and EI_min_ were higher in children receiving HU compared to children not receiving HU, and POS was lower. None of these results reached statistical significance.

When calculating multiple linear regressions controlling for age, %HbF and α-thalassaemia genotype, HU treatment was not found to be a significant predictor for any of the Oxygenscan-derived biomarkers.

### Sickle cell pain

In the two years prior to study inclusion, 48 children had experienced one or more painful crisis requiring a visit to the emergency department or resulting in hospital admission, and 43 children had no such episodes. There were no significant differences between these groups for El_max_, El_min_ or POS. A +1 SD change in EI_max_ was associated with an odds ratio (OR) of 1·4 for experiencing painful crises (P = 0·09), a +1 SD change in EI_min_ was associated with an OR of 1·3 for experiencing painful crises (P = 0·3), and a +1 mm Hg change in POS was associated with an OR of 1 for experiencing painful crises (P = 0·8). Results were similar when controlling for age, HU treatment, %HbF and α-thalassaemia genotype.

When examining outcomes for self-reported pain (daily/weekly/monthly/yearly/never), results were similarly negative. We found that a change of +1 SD in EI_max_ was associated with a proportional OR of 0·9 on level of self-reported pain (P = 0·5), a change of +1 SD in EI_min_ was associated with a proportional OR of 1·3 on level of self-reported pain (P = 0·1), and a change of +1 mm Hg in POS was associated with a proportional OR of 1 on level of self-reported pain (P = 0·08). Results were similar when controlling for age, HU treatment, %HbF and α-thalassaemia genotype.

### Transcranial Doppler velocities

**Fig 2:**
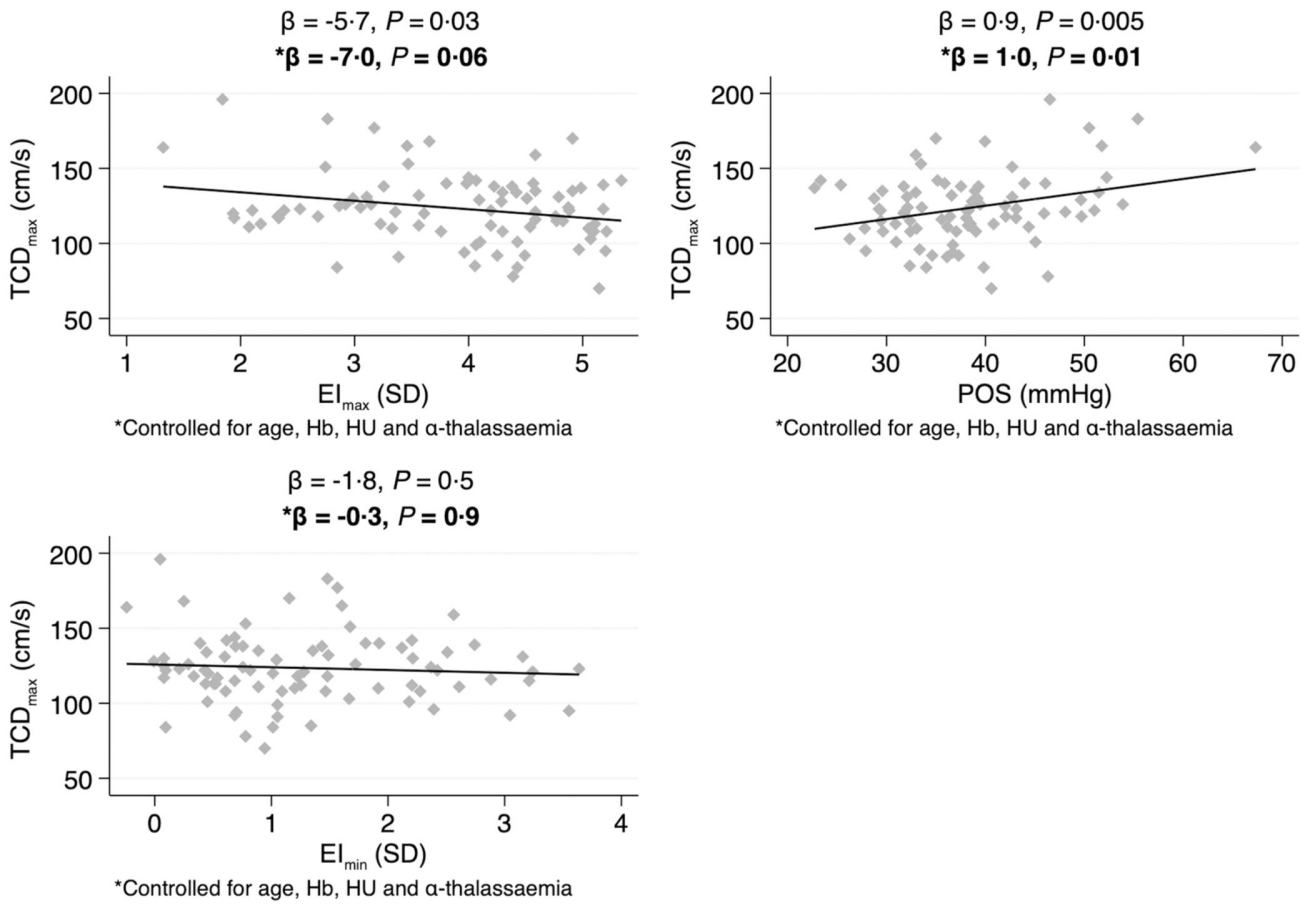
Linear relationship between maximum transcranial doppler velocities (TCD_max_) and Oxygenscan-derived biomarkers. EI, elongation index; Hb, haemoglobin; HU, hydroxyurea; POS, point of sickling; SD, standard deviation; TCD, transcranial doppler.

TCD measurements were available for 83 children. Linear regressions were calculated to predict TCD_max_ based on Oxygenscan-derived biomarkers (Fig 2). EI_max_ was negatively associated with TCD_max_, and results suggested an average decrease in TCD_max_ of — 5·7 cm/s per +1 SD increase in EI_max_ (*P* = 0·03). POS was positively associated with TCD_max_, and results suggested an average increase in TCD_max_ of +0·9 cm/s per +1 mm Hg increase in POS (P = 0·005). There was no significant association between EI_min_ and TCD_max_.

When calculating multiple linear regressions controlling for age, Hb, HU treatment and α-thalassaemia genotype, only results for POS remained significant. These suggested an average increase in TCD_max_ of +1 cm/s per +1 mm Hg increase in POS (*P* = 0·01).

## Discussion

Oxygen gradient ektacytometry is a functional assay developed to study RBC deformability in SCD. The Oxygenscan aims to describe how RBC deformability changes with oxygen tension, with the potential to identify individuals with SCD at increased risk of particular complications and assess responses to new and existing treatments. So far, only a limited number of studies have evaluated the method.

We conducted a study examining RBC deformability measured with the Oxygenscan in 91 children with SCA (HbSS) at King’s College Hospital in London, none of which had received any blood transfusions within three months of study recruitment. To the best of our knowledge, this study is the first to evaluate Oxygenscan-derived biomarkers in a homogeneous paediatric cohort of this size.

Previous studies have shown that the time between sample collection and sample analyses influences Oxygenscan results.^13^ Sample storage for >24 h can particularly affect EImax and EImin, even when stored at 4°C, whereas the POS seems to remain stable. Because our samples were shipped from London to Copenhagen overnight it was not feasible to analyse samples within 24 h. To best ensure that results were comparable across our study, samples were all treated in a standardised manner and kept at 4°C and measured between 24 and 48 h from sampling. Nevertheless, the delay in analysis causes an inherent limitation to our study.

As anticipated, we found a significant correlation between key Oxygenscan-derived biomarkers and %HbF, a well-known disease modifier in SCA. Both EImax and EImin demonstrated a moderate positive correlation with %HbF and POS demonstrated a moderate negative correlation with %HbF. These results, which are similar to those found by other research groups,^11,15^ illustrate how increasing levels of HbF are associated with slower rates of HbS polymerisation with improved erythrocyte deformability, and that the RBCs of individuals with high HbF levels can endure lower oxygen tensions before sickling begins.

Another well-recognised modifier of disease severity in SCA is α-thalassaemia.^19,20^ Individuals with co-inherited hetero- or homozygous α-thalassaemia typically present with fewer organ or life-threatening complications compared to individuals with no α-thalassaemia deletions, although the incidence of acute pain is unchanged or possibly increased. A recent study by Boisson *et al*.^21^ examined the impact of α-thalassaemia genotype on Oxygenscan parameters in adults and children with HbSS. When comparing results from 12 individuals with homozygous α-thalassaemia to those from 97 individuals with heterozygous α-thalassaemia or no α-thalassaemia deletions, they found a significant difference in EI_min_ and POS. Results for EI_max_, however, did not reach statistical significance. These results were not controlled for any confounding factors. In our study, only seven children were found to be homozygous for the α^-3^·^7kb^ deletion. In our unadjusted analysis, we found that EI_max_ increased significantly with the number of α-gene deletions, whereas EI_min_ values were lower in homozygous children compared to children who were heterozygous or had no deletions. Results for EI_min_ were not statistically significant and could be explained by the limited no of homozygous children. Despite the small sample size, we found co-inherited hetero- or homozygous α-thalassaemia to be a significant predictor of EI_max_, EI_min_ and POS, when controlling for age, %HbF and HU treatment. These results are similar to those of studies using osmotic gradient ektacytometry^22–24^ and suggest that RBCs of individuals with co-inherited α-thalassaemia demonstrate higher deformability, both at normoxia and at low oxygen tensions, as well as an increased tolerance to deoxygenation. Embury *et al*.^24^ found that the number of α-globin genes determines the distribution of DRBCs, hereby affecting the average deformability measured on the total RBC population, but does not modulate deformability in RBCs of similar density. Future studies examining Oxygenscan in SCA RBCs of different densities could be valuable in order to better understand sickling properties of specific cell populations at varying oxygen tensions, although the need for such cell separation would inevitably limit the use of the Oxygenscan in clinical practice.

Boisson et al.^16^ previously published findings of the effects of HU treatment on Oxygenscan-derived biomarkers in adults and children with SCA. In their study they compared results from 29 non-transfused adults and children with HbSS not receiving HU (mean age 24·1 years) to those from 45 adults and children (mean age 27·1 years) treated with HU. EI_min_ and EI_max_ were found to be higher in the HU group (*P* < 0·01), whereas POS was not significantly different between the groups. Results were not controlled for potential confounding factors, such as age or %HbF. In our study we did not find any significant difference in Oxygenscan-derived biomarkers in children treated with or without HU. This may reflect baseline differences in children taking or not taking HU, but also suggests that the effects of HU are predominantly mediated by increased HbF levels, with little or no other effects on red cell function. This is in keeping with experiments which found no improvement in haemolysis associated with HU use in mouse models of SCA, in which induction of HbF did not occur.^25^ Furthermore, Ballas *et al*.^26^ found that increasing levels of HbF improved RBC deformability measured with ektacytometry, irrespective of HU treatment and α-thalassaemia genotype. Rab et al.^14^ reported on Oxygenscan results in 15 individuals with SCD before and during HU treatment. They found a significant decrease in POS and an increase in EI_max_ and EI_min_ after three and six months of treatment. Although this is potentially a more accurate way of assessing treatment response, larger prospective studies are needed to determine effects of HU on Oxygenscan-derived biomarkers. Overall, there is not much evidence that the Oxygenscan provides any extra information on the effects of HU beyond that provided by measuring the %HbF.

Rab *et al*.^14^ recently reported Oxygenscan-derived biomarkers to be associated with the frequency of vasoocclusive crises (VOCs). They performed Oxygenscan analyses on samples from 80 children and 46 adults with varying SCD genotypes, comparing results between individuals who had experienced one or more VOCs in the past two years and individuals with no such episodes. All samples were taken at steady state. In the paediatric cohort, 52 children (65%) were treated with HU and 11 (14%) were on a chronic transfusion regime. Results suggested that POS was higher in children who had experienced VOCs compared to children with no VOCs (*P* = 0·0495) and EI_min_ was lower in children who had experienced VOCs (*P* = 0·02), whereas the difference for EI_max_ did not reach statistical significance. In the adult cohort, all three Oxygenscan-derived biomarkers differed significantly between individuals with or without a history of VOC. Although not highly significant, these results indicate that SCA RBCs in individuals with a history of painful crises tolerate lower oxygen tensions and are less deformable after deoxygenation. In contrast, older studies investigating RBC deformability in children and adults with SCA using ektacytometry found deformability to be positively associated with VOC frequency,^2,23,27^ and proposed that increased deformability in SCA causes an increase in RBC vascular adherence, thereby increasing rates of VOC. Our results, although not statistically significant, similarly suggest higher deformability (expressed by EI_max_ and EI_min_) to be associated with more frequent painful crises. In our study, eight children had EI_min_ values >0·4 while EI_max_ was <0·6, representing relatively ‘normal’ Oxygenscan results. In theory, such results should characterise children with few clinical complications. However, six of these children had experienced one or more painful crisis within the past two years, with one child having been admitted to hospital with pain four times within the past 12 months. Admittedly, pain is a difficult outcome to examine and multiple factors could have influenced the results. Nonetheless, there is currently little evidence that Oxygenscan-derived biomarkers can predict sickle cell pain and replace patient-reported outcomes in clinical and clinical-trial settings.

We found some evidence suggesting a negative linear relationship between TCD_max_ and POS. Whether this association merely reflects other factors known to influence TCD velocities is difficult to say. It is worth noting that we have conducted a number of comparison analyses in this study and that a multiple-comparison correction could arguably have been applied, in which case results for TCD_max_ would no longer have reached statistical significance.

Finally, an important limitation to consider is that the Oxygenscan measures deformability of the total RBC population. The presence of dense and irreversibly sickled cells influences the average deformability of the RBCs,^5,24^ explaining why the percentage of DRBCs correlates negatively with EI_max_.^10^ However, the Oxygenscan only assesses sickling properties in cells which can change their deformability in response to changes in oxygen tension, and so does not examine the function of irreversibly sickled cells or other damaged cells with reduced deformability. This may be one reason why results do not correlate well with clinical complications or change predictably with HU treatment.

## Conclusion

We found that the Oxygenscan-derived biomarkers EI_max_, EI_min_ and POS all correlate with the known modifiers of disease severity in SCA, %HbF and co-inherited α-thalassaemia. Furthermore, there seems to be some association between the POS and TCD velocities. We failed, however, to find any independent predictive value of the assay in the pivotal clinical outcome measure of pain, as well as other important parameters such as HU treatment. Although the Oxygenscan remains an intriguing tool for basic research, our results question whether it provides any additional information in predicting the clinical course in children with SCA, beyond measuring known markers of disease severity.

## Figures and Tables

**Table I:** Baseline characteristics of study population (*n* = 91 unless otherwise indicated).

	Mean (range)
Age (years)	8·4 (7 months-16 years)
Girls, *n* (%)	45 (49·5%)
HU treatment, *n* (%)	43 (47·3%)
**α**-thalassaemia (3·7-kb deletion), *n* (%)	
Not detected	47 (51·6%)
Heterozygous	33 (36·3%)
Homozygous	7 (7·7%)
Not performed	4 (4·4%)
G6PD, *n* (%)	
Normal activity	77 (84·6%)
Deficiency	9 (9·9%)
Not performed	5 (5·5%)
Oxygenscan-derived biomarkers	
EI_max_	0·426 (0·142–0·574)
EI_min_	0·177 (–0·031–0·492)
POS (mm Hg)	37·4 (22·1–67·3)
Laboratory values	
HbF (%), *n* = 86	14·2 (1·3–36·4)
Hb (g/l), *n* = 89	87·5 (62–116)
Bilirubin (μmol/l), *n* = 88	37 (10–138)
LDH (iu/l), *n =* 72	564 (312–977)
Reticulocytes (%), *n* = 89	12·5 (2·7–36)
Clinical outcomes	
≥1 painful crisis in the past two years, *n* (%)	48 (52·7%)
Self-reported pain in the past 12 months, *n* (%)	
Never	18 (19·8%)
Yearly	15 (16·5%)
Monthly	41 (45%)
Weekly	14 (15·4%)
Daily	3 (3·3%)
TCD_max_ (cm/s), *n* = 83	123·5 (70–196)

EI, elongation index; G6PD, glucose-6-phosphate dehydrogenase; Hb, haemoglobin; HbF, haemoglobin F; HU, hydroxyurea; LDH, lactate dehydrogenase; POS, point of sickling; TCD, transcranial Doppler.

**Table II:** Oxygenscan-derived biomarkers according to **α**-thalassaemia genotype (*n* = 87).

	No **α**-thalassaemia deletions (mean ± SD)	Heterozygous **α**-thalassaemia (mean ± SD)	Homozygous **α**-thalassaemia (mean ± SD)	
EI_max_	0·398 ± 0·112	0·448 ± 0·102	0·480 ± 0·069	*P* = 0·04
EI_min_	0·150 ± 0·111	0·210 ± 0·134	0·128 ± 0·136	*P* = 0·07
POS (mm Hg)	40·1 ± 8·9	34·4 ± 6·4	35·0 ± 6·0	*P* = 0·006

EI, elongation index; POS, point of sickling; SD, standard deviation.

**Table III:** Oxygenscan-derived biomarkers in children with and without G6PD deficiency (*n* = 86).

	G6PD deficiency (*n* = 9) (mean ± SD)	Normal G6PD activity (*n* = 77) (mean ± SD)	
EI_max_	0·431 ± 0·133	0·423 ± 0·107	*P* = 0·8
EI_min_	0·242 ± 0·184	0·166 ± 0·122	*P* = 0·1
POS (mm Hg)	35·8 ± 5·8	37·7 ± 8·4	*P* = 0·5

EI, elongation index; G6PD, glucose-6-phosphate dehydrogenase; SD, standard deviation; POS, point of sickling.

**Table IV:** Correlation between oxygenscan-derived biomarkers, haemoglobin and markers of haemolysis.

	Haemoglobin (g/l) (*n* = 88)	Bilirubin (μmol/l) (*n* = 88)	LDH (iu/l) (*n* = 72)	Reticulocytes (%) (*n =* 89)
EI_max_	*r* = 0·5, *P* < 0·001	*r* = −0·5, *P* < 0·001	*r* = −0·4, *P* < 0·001	*r* = −0·3, *P* = 0·004
EI_min_	*r* = 0·1, *P* = 0·4	*r* = −0·3, *P* = 0·004	*r* = −0·2, *P* = 0·08	*r* = −0·1, *P* = 0·3
POS (mm Hg)	*r* = −0·4, *P* < 0·001	*r* = 0·3, *P* = 0·007	*r* = 0·3, *P* = 0·005	*r* = 0·2, *P* = 0·09

EI, elongation index; LDH, lactate dehydrogenase; POS, point of sickling; *r*, correlation coefficient.

**Table V:** Oxygenscan-derived biomarkers in children treated with and without hydroxycarbamide (*n* = 91).

	−HU treatment (mean ± SD)	+HU treatment (mean ± SD)	
EI_max_	0·408 ± 0·120	0·446 ± 0·089	*P* = 0·09
EI_min_	0·159 ± 0·137	0·197 ± 0·116	*P* = 0·2
POS (mm Hg)	38·5 ± 9·3	36·2 ± 6·5	*P* = 0·2

EI, elongation index; HU, hydroxyurea; SD, standard deviation; POS, point of sickling.
